# Case Report: Purple urine bag syndrome in woman with neurogenic bladder

**DOI:** 10.12688/f1000research.19393.1

**Published:** 2019-06-27

**Authors:** Senohadi Boentoro, Nugroho Budi Utomo

**Affiliations:** 1 Department of Urology, Faculty of Medicine, Universitas Indonesia, Dr. Cipto Mangunkusumo Hospital, Jalan Diponegoro 71, Jakarta, 10430, Indonesia; 2 Gatot Soebroto Army Hospital, Jalan Abdul Rahman Saleh 24, Jakarta, 10410, Indonesia

**Keywords:** purple urine bag syndrome, neurogenic bladder, elderly woman, indwelling catheter

## Abstract

Purple urine bag syndrome (PUBS) is a rare phenomenon in patients that is associated with the use of a long-term/indwelling urinary catheter. The purple color results from indigo and indirubin, accumulated from bacteria-mediated tryptophan conversion. High risk patients include: the elderly; women; immobilized patients; patients with an indwelling catheter, chronic constipation, alkaline urine or poor hygiene; and those with catheter bags and tubes made of certain types of plastic. We reported PUBS in an elderly woman with an indwelling catheter and chronic constipation which, to our knowledge, was the first case in our hospital. The patient underwent urinary catheter change and received intravenous ciprofloxacin, following which the urine returned to a yellow color and the patient was discharged. This case report describes the diagnosis, management and also strategies for the prevention of PUBS in Gatot Soebroto Army Hospital, Indonesia.

## Introduction

Purple urine bag syndrome (PUBS) is a clinical phenomenon associated with urinary tract infections (UTIs) due to long-term use of catheters, usually occurring in elderly patients. PUBS was first reported by Barlow and Dickson in 1978 ^1^. This phenomenon can cause panic for patients and caregivers because the color changes from yellow to purple. This change is known to occur only in the urine bag, while the color of urine does not actually change to purple ^2, 3^. Hereby, we report this rare phenomenon in an elderly woman with neurogenic bladder which, to our knowledge, was the first PUBS case in Gatot Soberoto Army Hospital, Indonesia and the first published case of PUBS from Indonesia.

## Case presentation

**Figure 1.**
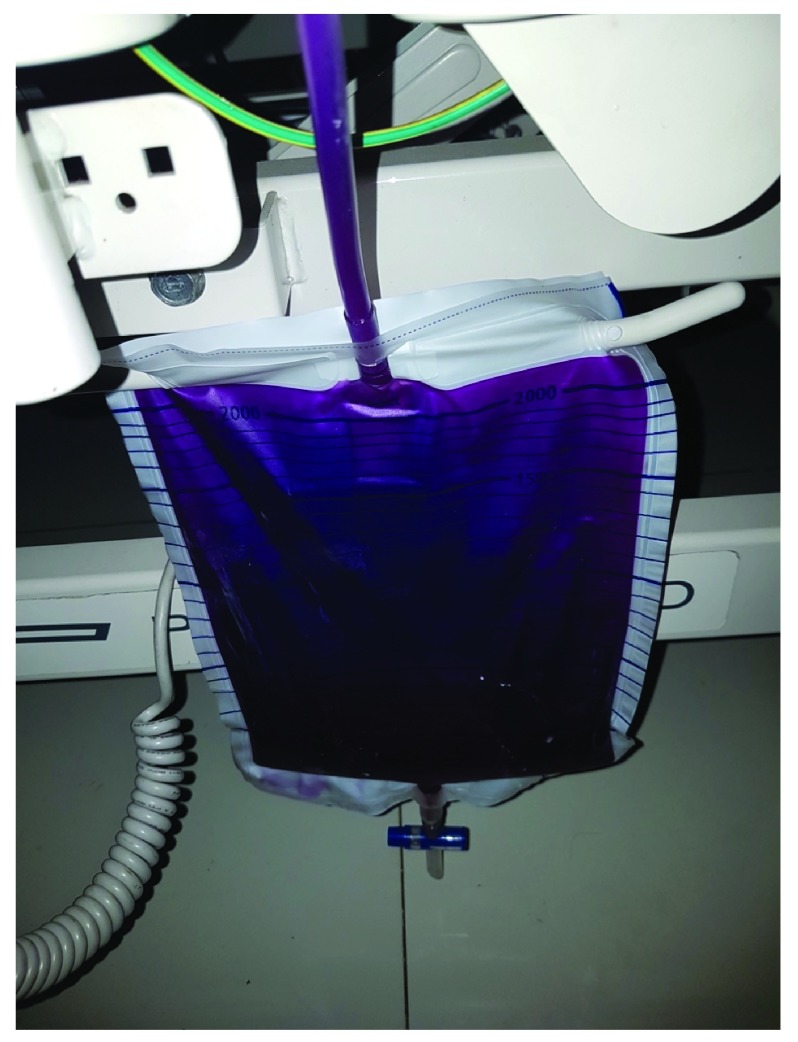
Urine bag with purple urine.

A 64-year-old retired Southeast Asian woman was admitted to Gatot Soebroto Army Hospital, Jakarta because of fever for two days and had a consultation with a urologist because the urine in her urine bag had changed color to purple ( Figure 1). This discoloration was first noticed by her daughter at home approximately three hours before the hospital admission.

The patient was known to have neurogenic bladder caused by a spinal cord injury. She had undergone several rehabilitative treatment sessions and had been using a catheter for three months. The patient had also suffered from constipation for three months previously. At the consultation, no abnormalities were found in the flank region. The patient’s bladder was palpated to ensure that it was empty, and no masses or pain were found in the suprapubic region. There were no abnormalities observed upon physical examination except motor weakness (paraparesis) in both lower extremities. The patient had a 16-Fr indwelling catheter and purple urine production of 1,350 ml/24 hours.

Laboratory blood tests, including complete blood count, renal function test, liver function test and electrolyte levels, showed an increase in leukocytes (12,680/μL [normal range 5,000-10,000/μL]), with other test results within normal limits. Urinalysis, including macroscopic and microscopic analysis (test for color, sedimentation, erythrocytes, urinary casts, sedimentation, epithelial cells, crystals, bacteria, specific gravity, pH, albumin, glucose, bilirubin, urobilinogen, blood, nitrite and leucocyte esterase), showed urinary alkalosis (pH 8.5 [normal range 4.5-8.0]), nitrite positive (+2), leucocyte esterase positive (+2), with other test results within normal limits. Urine culture for aerobic bacteria, anaerobic bacteria and yeast was carried out before the administration of antibiotics. *Escherichia coli* culture results showed significant growth (>100,000/mL after 24 hours), which was sensitive to ciprofloxacin, nitrofurantoin and amikacin.

**Figure 2.**
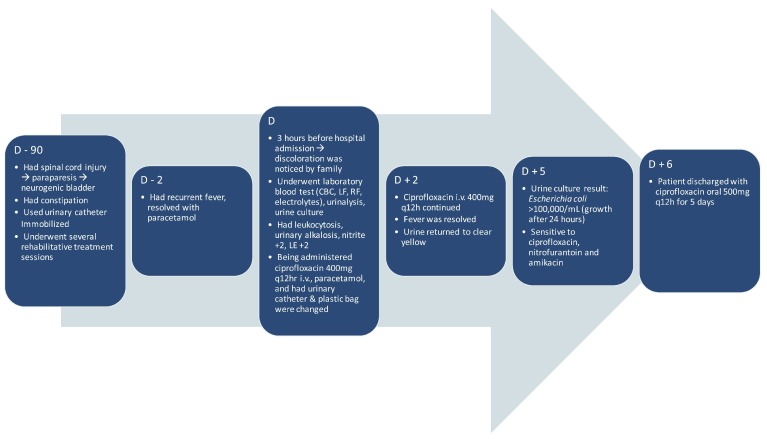
Timeline of the course of illness.

The patient was then given intravenous ciprofloxacin (400 mg q12h) and antipyretic (paracetamol 500mg when needed). The catheter and plastic bag were changed, and changes to the urine color occurred. After the administration of ciprofloxacin therapy (400mg q12h) for two days, the fever was resolved, and the urine returned to a clear yellow color. To avoid the development of antimicrobial resistance, ciprofloxacin (400mg q12h) was continuously given for a total of seven days. The patient was discharged afterwards and there was no further follow-up. The course of illness is shown as a timeline in Figure 2.

## Discussion

Although UTIs can occur at various age ranges, PUBS is commonly found in the elderly, especially in women, patients with chronic catheter use, patients with chronic constipation or for UTIs associated with sulfate/phosphatase production ^2^. The prevalence of UTIs is estimated to be around 8.3-42,1% in hospitalized patients with long-term catheter use ^4^. Around 9.8% of hospitalized patients with long-term catheter use experience PUBS ^2^.

Most PUBS patients use long-term catheters because of disturbances to mobilization, such as patients who use wheelchairs or are confined to bed rest ^5^. The mechanism of how constipation causes PUBS is through changes in intestinal motility. Constipation can prolong tryptophan transit, resulting in increased levels of indoxyl sulfate in the urine ^3, 5^. A short urethra in women is a UTI predisposing factor, which is also seen in PUBS patients ^5^. Dehydration is also considered as one of the risk factors for PUBS, due to increased indigo concentration and indirubin in the urine ^4^. In addition, other PUBS risk factors are the use of catheters made of polyvinyl chloride plastic ^5^. There is an increased risk of PUBS in patients with renal failure, associated with a decrease in indoxyl sulfate clearance, so bacteria produce more indigo and indirubin ^4^.

**Figure 3.**
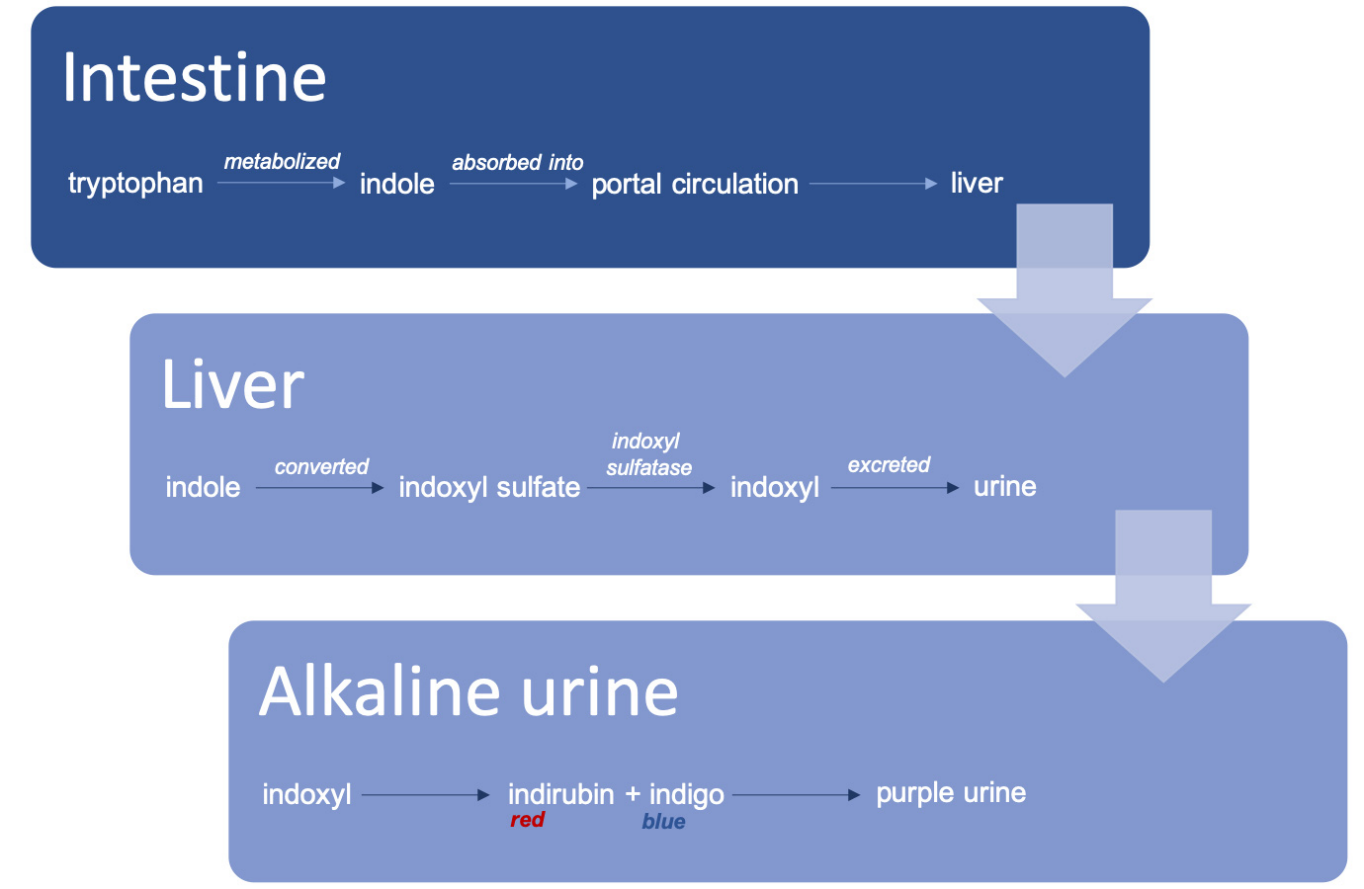
Pathophysiology of purple urine bag syndrome (PUBS).

The purple etiology of PUBS comes from the mixture of red (indirubin) and blue (indigo) chemicals resulting from tryptophan metabolism. Bacteria in the intestine metabolize tryptophan, producing indoles that are then absorbed into the portal circulation. In the liver, indoxyl sulfate is produced from indole conversion. Indoxyl sulfate is digested by bacteria, which produce indoxyl sulfatase and convert it to indoxyl. Then indoxyl is excreted through urine. In alkaline urine, indoxyl changes to indirubin (red) and indigo (blue), and the mixture of these two colors produces purple ( Figure 3).

Other causes of purple coloration are conjugated bile acids or steroids. The conversion of tryptophan to indole increases with excessive growth of colon bacteria in patients with chronic constipation. Both this change in urine composition and chronic constipation cause the prevalence of PUBS to be higher in patients who have constipation and long-term use of urinary catheters ^2^.

Some bacteria known to be associated with PUBS are *Providencia stuartii, Providencia rettgeri, Klebsiella pneumoniae, Proteus sp., Escherichia coli, Enterococcus sp., Morganella morganii,* and *Pseudomonas aeruginosa* ^2^. In patients with long-term catheter use, *P. mirabilis* is a particular problem for medical care. Urinary catheters are considered one of the main risk factors for hospital-acquired infections based on previous prevalence studies. Therefore, the unnecessary use of urinary catheters should be avoided to reduce UTI rates.

Management of PUBS consists of improvements to hygiene, including replacement of urine catheter, management of constipation and eradication of the UTI. Control and prevention of catheter-associated UTIs should be an important element of the medical care for these patients ^5^. The most commonly given antibiotic for PUBS is ciprofloxacin (quinolone), which is considered appropriate empirical therapy ^4^. Some other antibiotic options that can be chosen are piperacillin/tazobactam, ticarcillin/clavulanate, ampicillin/sulbactam, ceftazidime, cefepime, levofloxacin, norfloxacin, moxifloxacin, meropenem and ertapenem. These antibiotics are effective against gram-negative bacteria ^5^. Based on suspicion, antibiotics can be started before the results of urinalysis and urine culture are available ^5^. In immunocompromised patients and persistent PUBS, antibodies can be given intravenously ^4^. In previous studies, PUBS was suggested to be a sign of Fournier gangrene in immunocompromised patients and so more attention is needed to these patients. Non-plastic urine bags as a prevention method for PUBS can also be taken into consideration.

As the first reported case in our hospital, we hope this study could help in avoiding the underdiagnosis of PUBS by medical staff, especially in our hospital and in Indonesia. The limitation of this study was that there was no follow-up after patient discharge.

## Conclusion

Although PUBS is not a dangerous clinical condition, the sudden discoloration of urine to purple can cause panic for patients and families. No special management for patients with PUBS is needed apart from appropriate antibiotics according to culture results, good catheter hygiene when using catheters for a long period of time, replacement of catheters and urine bags on time and treatment of constipation. This phenomenon of PUBS needs to be known, not only by urologists, but also by other doctors and medical personnel, so that panic does not arise, and personnel can help calm patients and families. In managing patients, especially elderly patients and women, with long-term catheterization and chronic constipation, we should be aware of the risk of developing UTIs and ensure good catheter hygiene to avoid any preventable complications, including PUBS.

## Data availability

All data underlying the results are available as part of the article and no additional source data are required.

## Consent

Written informed consent for publication of their clinical details and/or clinical images was obtained from the patient.
