# Supplementary material for: Case Report: Purple urine bag syndrome in woman with neurogenic bladder
Source: F1000Res. 2019 Jun 27;8:979. [Version 1] doi: 10.12688/f1000research.19393.1 (PMC6720032; doi:10.12688/f1000research.19393.1)
Supplement: Peer review history

# Peer review: Reviewer response for version 1

**DOI:** 10.5256/f1000research.21259.r52318

**Published:** 2019-09-02

**Authors:** Kannan Rajendran

**Affiliations:** 1 Department of General Medicine, Saveetha Medical College and Hospital, Saveetha University, Chennai, Tamil Nadu, India

- The hospital and country name can be avoided. 
- The article is very well written.
- Good photo.
- Description is clear.
- It can be accepted for publication.  
- The editing part also acceptable.
- PUBS nowadays is becoming more common, it was one of the rare entities earlier. We have reported twice from our hospital.

I confirm that I have read this submission and believe that I have an appropriate level of expertise to confirm that it is of an acceptable scientific standard.

# Peer review: Reviewer response for version 1

**DOI:** 10.5256/f1000research.21259.r52319

**Published:** 2019-08-23

**Authors:** Chinmay Patel

**Affiliations:** 1 Division of Nephrology, Kentucky College of Osteopathic Medicine, Pikeville Medical Center, University of Pikeville, Pikeville, KY, USA

This is a well written manuscript. The authors have nicely described the case, pathogenesis of Purple Urine Bag Syndrome (PUBS)  and followed up with excellent management points.

 PUBS is a rare but important phenomenon seen in elderly patients with UTI and indwelling foley catheters. Such patients can be nonverbal and asymptomatic with purple urine bag being the only sign of UTI!

 Doctors and other health care professionals should be familiar with this phenomenon due  its morbidity. 

 I recommend this manuscript for indexing.

I confirm that I have read this submission and believe that I have an appropriate level of expertise to confirm that it is of an acceptable scientific standard.
